# 4.1 ENHANCING EARLY PSYCHOSIS TREATMENT USING SMARTPHONE TECHNOLOGY: INTEGRATION OF A MOBILE HEALTH PLATFORM IN FOUR EARLY PSYCHOSIS PROGRAMS

**DOI:** 10.1093/schbul/sby014.008

**Published:** 2018-04-01

**Authors:** Laura Tully, Ana-Maria Iosif, Lauren Zakskorn, Divya Kumar, Kathleen Nye, Aqsa Zia, Jennifer Denton, Katherine Pierce, Taylor Fedechko, Tara Niendam

**Affiliations:** University of California, Davis

## Abstract

**Background:**

Mobile health applications offer ecologically valid, data-rich methods of modeling daily symptoms and functioning, which could inform treatment delivery and facilitate early intervention in individuals with psychosis. To date, most studies evaluate adoption of technology independent of care providers. However, successful implementation and long-term adoption of mobile technology likely also requires integration into outpatient settings as an add-on tool to enhance treatment. We implemented a smartphone “app” plus clinician Dashboard as an add-on treatment tool in the UC Davis Early Psychosis (EP) Programs and tested feasibility, validity, and predictive utility of symptom tracking via the app as part of EP care. A subsequent pilot study examined barriers to implementation within two additional community outpatient settings in Northern California.

**Methods:**

Study 1 implemented the platform within the UC Davis EP Programs. For up to 14 months, EP clients completed daily and weekly surveys examining mood, symptoms, and treatment relevant factors via the app, as well as monthly in-person clinical assessments using the BPRS. Clinicians discussed symptom ratings and surveys during treatment sessions using the Dashboard. We examined client enrollment and survey completion to determine feasibility, and relationships between BPRS and weekly symptom ratings to evaluate validity of self-report symptom data collected via the app. Analysis of predictive utility determined if weekly self-report symptoms predicted symptom exacerbations 2 weeks later. Study 2 expanded recruitment to 2 additional community-based EP outpatient clinics. EP clients and their clinicians used the platform as part of care for 5 months and filled out satisfaction surveys at study-end regarding usability of the platform. Rate of survey completion in the absence of financial incentives was examined to determine real-world implementation of the platform.

**Results:**

For study 1, 76 clients enrolled and remained in the study for an average of 183 days (SD=88). Survey completion rates remained high over the course of the study (weekly surveys: 77%; daily surveys: 69%) and were not significantly impacted by baseline symptom severity or length of time in the study. Weekly survey positive and depression/anxiety symptoms were significantly associated with BPRS positive (p<0.001) and BPRS depression/anxiety symptoms (p< 0.001) respectively. EP clients reported high satisfaction with the platform and endorsed continue use of the app if it was made available as part of their treatment. For Study 2, 61 EP clients and 20 clinicians enrolled; 41 EP clients and 20 clinicians participated for 5 months. The majority of EP clients (66%) and clinicians (85%) who completed satisfaction surveys reported a desire to continue to use the platform as part of care. Six (15%) clients and 3 providers (23%) stated that technological glitches impeded their use of the platform.

**Discussion:**

These data support the validity and acceptability of implementing smartphone-based assessment of symptoms in community-based EP care. Specifically, results indicate that assessing positive and depression/anxiety symptoms using weekly self-report surveys via smartphone is comparable to gold-standard clinician-led assessments. This approach may be a valid method of monitoring fluctuations in positive and depression/anxiety symptoms in EP populations to anticipate symptom exacerbations. However, solutions to logistical barriers such as technical challenges and clinician engagement with technology are necessary for widespread adoption across EP care.

